# Identifying Microbiota Signature and Functional Rules Associated With Bacterial Subtypes in Human Intestine

**DOI:** 10.3389/fgene.2019.01146

**Published:** 2019-11-15

**Authors:** Lijuan Chen, Daojie Li, Ye Shao, Hui Wang, Yuqing Liu, Yunhua Zhang

**Affiliations:** ^1^College of Animal Science and Technology, Anhui Agricultural University, Hefei, China; ^2^School of Medicine, Huaqiao University, Quanzhou, China; ^3^Anhui Province Key Laboratory of Farmland Ecological Conservation and Pollution Prevention, School of Resources and Environment, Anhui Agricultural University, Hefei, China

**Keywords:** gut microbiome, bacteria feature, pattern, rule, multi-class classification

## Abstract

Gut microbiomes are integral microflora located in the human intestine with particular symbiosis. Among all microorganisms in the human intestine, bacteria are the most significant subgroup that contains many unique and functional species. The distribution patterns of bacteria in the human intestine not only reflect the different microenvironments in different sections of the intestine but also indicate that bacteria may have unique biological functions corresponding to their proper regions of the intestine. However, describing the functional differences between the bacterial subgroups and their distributions in different individuals is difficult using traditional computational approaches. Here, we first attempted to introduce four effective sets of bacterial features from independent databases. We then presented a novel computational approach to identify potential distinctive features among bacterial subgroups based on a systematic dataset on the gut microbiome from approximately 1,500 human gut bacterial strains. We also established a group of quantitative rules for explaining such distinctions. Results may reveal the microstructural characteristics of the intestinal flora and deepen our understanding on the regulatory role of bacterial subgroups in the human intestine.

## Introduction

Gut microbiome refers to the integral microflora that is located in the human intestine and has symbiosis with human beings (Arumugam et al., 2011; Yatsunenko et al., 2012). According to recent publications, the identified microflora in the human intestine contains tens of trillions of microorganisms including bacteria, fungi, protists, archaea, and viruses (Yatsunenko et al., 2012). Among different subgroups of microorganisms, bacteria are the most significant subgroup that contains unique and functional species between 300 and 1000 (Barcenilla et al., 2000; Chadchan et al., 2019). More than 60% of all microorganisms can be clustered into different bacterial subgroups. In different sections of the human intestine, the species distributions of bacteria are quite different (Reichardt et al., 2014). For instance, in the gut, almost all the identified bacteria are anaerobes; however, in the cecum, aerobic bacteria, another subgroup of bacteria, are predominant (Wells et al., 1987; Kelly et al., 2004). Such distribution patterns of bacteria in the human intestine not only reflect the different microenvironments in different sections of the intestine but also indicate that bacteria may have their unique biological functions corresponding to their proper regions of the intestine. The symbiosis of human beings and bacterial subgroups/clusters maintains the stability of the intestinal microenvironment (Arumugam et al., 2011; Yatsunenko et al., 2012).

In general, the biological functions of symbiotic gut bacteria can be summarized into three major aspects: intestine immune regulation (Kelly et al., 2005), nutrition metabolism regulation (Ramakrishna, 2013), and regulation of gut–brain axis (Foster and McVey Neufeld, 2013; Plummer et al., 2013). First, the gut bacteria can initiate and activate the humoral and adaptive immune responses in the specific region of the gut (Slack et al., 2009; Bunker et al., 2015). As one of the major subgroups of immune response-associated processes in the intestinal immune system, cytokine-associated biological processes are important; different subgroups of gut bacteria have been confirmed to increase different subgroups of cytokines (Atarashi et al., 2013; Schirmer et al., 2016). In addition, most bacteria, such as filamentous bacteria, can activate the musical immune responses, indicating that different subgroups of bacteria can have different biological contributions to immune regulatory processes (Wu et al., 2010). Different subgroups of bacteria also contribute to the digestion and absorption of nutrients through specific nutrition-associated biological functions. For instance, saccharolytic fermentation is a specific fermentation process that helps synthesize unique subtypes of short-chain fatty acids, which are required by various organs, such as the brain, liver, and kidney, and cannot be synthesized independently (Miller and Wolin, 1979; Windey et al., 2012). Different subgroups of gut bacteria contribute to the manufacture of different nutrient subtypes (Windey et al., 2012). Thus, the collaborative contribution of different gut bacterial subgroups can maintain the nutrition supply and physical health of human beings. Importantly, the direct relationship between the gut bacteria and the central nervous system, known as the gut–brain axis, has been confirmed in recent studies (Ghaisas et al., 2016; Kohler et al., 2016). Early in 2004, an independent experiment confirmed that germ-free mice, which do not have gut microbiome, exhibited improved hypothalamic–pituitary axis response compared with normal controls (Riediger et al., 2004). This study directly confirms that the gut microbiomes have potential causal effects on the central nervous system.

Bacterial distribution in the human intestine is significantly diverse and exerts various biological effects on human health. However, describing the functional differences between the bacterial subgroups and their distributions in different individuals is difficult using traditional computational approaches. Therefore, we attempted to introduce four effective sets of features from four independent databases, namely, the Antibiotic Resistance Genes Database (ARGD) (Liu and Pop, 2009), the Comprehensive Antibiotic Resistance Database (CARD) (McArthur et al., 2013; Jia et al., 2017), the Virulence Factor Database (VFDB) (Liu et al., 2019), and Kyoto Encyclopedia of Genes and Genomes (KEGG) database (Kanehisa, 2002; Tanabe and Kanehisa, 2012). The combination of features may comprehensively describe the biological functions of different bacterial subgroups and screen their most critical differences. In the present study, using the dataset established by a systematic analysis on the gut microbiome from approximately 1500 human gut bacteria phyla (Zou et al., 2019), we presented a novel computational approach to identify the potential distinctive features among bacterial subgroups and established a group of quantitative rules for explaining such distinctions. We only focused on three bacterial subgroups, namely, Actinobacteria, Bacteroidetes, and Firmicutes, due to the quantitative characteristics of the sequencing data. Our results may reveal the microstructural characteristics of the intestinal flora and deepen our understanding on the regulatory role of bacterial subgroups in the human intestine.

## Materials and Methods

### Datasets

We downloaded the functional annotations of human gut bacteria from the China National GeneBank under Project ID: CNP0000126 (https://db.cngb.org/search/project/CNP0000126/) (Zou et al., 2019). Each human gut bacteria were encoded with 342 Antibiotic Resistance Genes Database (ARDB) annotation features, 259 CARD annotation features, 243 KEGG annotation features, and 149 VFDB annotation features (a total of 993 features). We analyzed three human gut bacteria phyla with number of strains greater than 100, namely, 235 Actinobacteria, 447 Bacteroidetes, and 796 Firmicutes. Fusobacteria with six strains and Proteobacteria with 36 strains were excluded. The goal was to find the functional difference among different human gut bacterial phyla.

Features from different databases have their independent biological significance. The first database (ARDB) was built up to provide a basic summary for antibiotic resistance and facilitate the identification and annotation of novel drug resistance associated genes (Liu and Pop, 2009). Features in such database describes the gene ontology, COD&COG taxonomy, KEGG pathway information (McArthur et al., 2013; Jia et al., 2017), and mutation resistance information of all the annotated genes (Liu and Pop, 2009). Using such features, we can easily describe the biological functions of effective genes and the potential pathogenic effects of specific mutations, classifying mutant and wild-type genes into different types (Liu and Pop, 2009). As for the second database, CARD, it summarizes all the characterized, peer-reviewed resistance determinants and associated antibiotics based on Antibiotic Resistance Ontology (ARO) and AMR gene detection models (McArthur et al., 2013; Jia et al., 2017). Features of such database mainly focused on the description of drug resistance characteristics of different microbial strains (McArthur et al., 2013; Jia et al., 2017). Deferentially, the next database named as VFDB (Liu et al., 2019) turns out to be an integrated and comprehensive online resource for bacterial pathogenic analysis. Features from such databases describe the virulence factors and potential pathogens of various microbial types (Liu et al., 2019). As for the last database, as we have mentioned above, KEGG database (McArthur et al., 2013; Jia et al., 2017) mainly focuses on the functional description of potential microbial genes. Features of such database describe the unique functional characteristics.

### Feature Ranking

Of the extracted 993 features from different sources, some features were redundant and not informative. To select the important features that contribute most to the classification tasks, we applied Monte Carlo feature selection (MCFS) (Cai et al., 2018; Chen et al., 2018a; Pan et al., 2018; Chen et al., 2019a; Chen et al., 2019c; Chen et al., 2019e; Li et al., 2019; Pan et al., 2019a; Pan et al., 2019b) to analyze these features and rank them according to their importance. MCFS is a supervised feature selection method based on multiple decision trees (Draminski et al., 2008). MCFS first generates *s* bootstrap sample sets and *m* feature subsets from the original data. A decision tree is grown for each combination of the bootstrap set and feature subset. Accordingly, *t*×*m* trees are constructed in total and used to calculate relative importance (RI) score for each feature with the assumption that the important features should be frequently involved in many growing decision trees. For each feature, RI score is calculated based on the following components: 1) number of splits involved in all nodes of *t*×*m* trees; 2) information gain by each split; and 3) classification accuracies of individual decision trees. Its calculation formula is as follows:

(1) $$RI_{g}={\sum_{\tau =1}^{t\times m}\left(wAcc\right)}^{u}\sum_{n_{g}(\tau )}IG(n_{g}(\tau )){\left(\frac{\text{no}\text{. in }n_{g}(\tau )}{\text{no}\text{. in }\tau }\right)}^{v}$$

where *IG*(*n* *_g_*(τ)) stands for the gain information of node *n* *_g_*(τ), (no.in *n* *_g_*(τ)) the number of samples in node *n* *_g_*(τ), no.in τ the number of samples in tree τ, *wAcc* the weighted accuracy of decision tree τ. *u*, and *v* represent two regular factors, which were all set to one in this study. After obtaining the RI score of each feature, all features were ranked by the decreasing order of their RI scores. MCFS was implemented and downloaded at http://www.ipipan.eu/staff/m.draminski/mcfs.html.

### Incremental Feature Selection

After ranking the input features by using MCFS, we determined whether all these features are necessary for classifying Actinobacteria, Bacteroidetes, and Firmicutes. We applied incremental feature selection (IFS) (Zhang et al., 2015a; Zhang et al., 2015b; Zhou et al., 2015; Chen et al., 2017b; Chen et al., 2017c; Liu et al., 2017; Chen et al., 2018b; Zhang et al., 2018; Chen et al., 2019d; Wang and Huang, 2019) with a classifier to the ranked features and selected the discriminate features with the best performance. Basing on the ranked features from MCFS, we constructed a series of feature subsets with step 1, e.g., the first feature subset has the top 1 feature, and the second subset has the top 1 and 2 features. For each feature subset, we trained a classifier on the samples consisting of features from the feature subset and evaluated the classification performance by 10-fold cross-validation. After running the process for all feature subsets, we selected the feature subset with the best performance (i.e., highest Matthews correlation coefficient); this feature subset was called the optimum feature subset.

### Rule Learning

Many different supervised classifiers, including black-box and interpretable rule-based methods, exist. Black-box methods cannot explain their predictions in a manner that humans can understand, and rule-based methods can supply classification reasons in a way understandable to humans. In this study, we used an interpretable rule-based classification method with repeated incremental pruning to produce error reduction (RIPPER) (Cohen, 1995; Li et al., 2019; Pan et al., 2019a) (i.e., Jrip algorithm) to classify the samples from three bacterial groups, namely, Actinobacteria, Bacteroidetes, and Firmicutes. In addition, a rule usually consists of if-then statement; simply put, if conditions A and B are met, then we make a certain prediction of yes or no. RIPPER is a greedy method for learning classification rules. This method first generates a good rule covering some samples in the training set. These covered samples are removed, and the remaining training set is used for the next rule. This process of rule generation is repeated until all samples are covered by the learned rules or predefined stop conditions are met. Lastly, the learned rules are further pruned using reduced error pruning.

To quickly implement the RIPPER algorithm mentioned above, a tool “JRip” in Weka (Witten and Frank, 2005) was directly employed in this study. For convenience, its default parameters were used.

### Performance Measurement

We used RIPPER as a multiclassification method to classify samples from Actinobacteria, Bacteroidetes, and Firmicutes. The 10-fold cross-validation was adopted for performance evaluation (Huang et al., 2009; Huang et al., 2010; Cai et al., 2012; Chen et al., 2013; Zhang et al., 2015a; Zhao et al., 2018; Zhang et al., 2019; Zhao et al., 2019), and the performance measurements should be appropriate for multiclass classification. Several measurements were employed in this task. They can be divided into two categories. The first measurement category was for each phylum, such as individual accuracy, precision, recall (same as individual accuracy), and Matthews correlation coefficient (MCC) (Matthews, 1975). The other measurement category fully evaluate the performance of the classification method, including overall accuracy and MCC in multi-class (Gorodkin, 2004), as detailed in previous works (Chen et al., 2017a; Li et al., 2018; Chen et al., 2019b; Chen et al., 2019c; Cui and Chen, 2019; Pan et al., 2019a; Pan et al., 2019b). Because MCC in multi-class is widely accepted to be a balanced measurement even if the dataset is of great imbalance, it was selected as the key measurement in our study.

## Results

**Figure 1:**
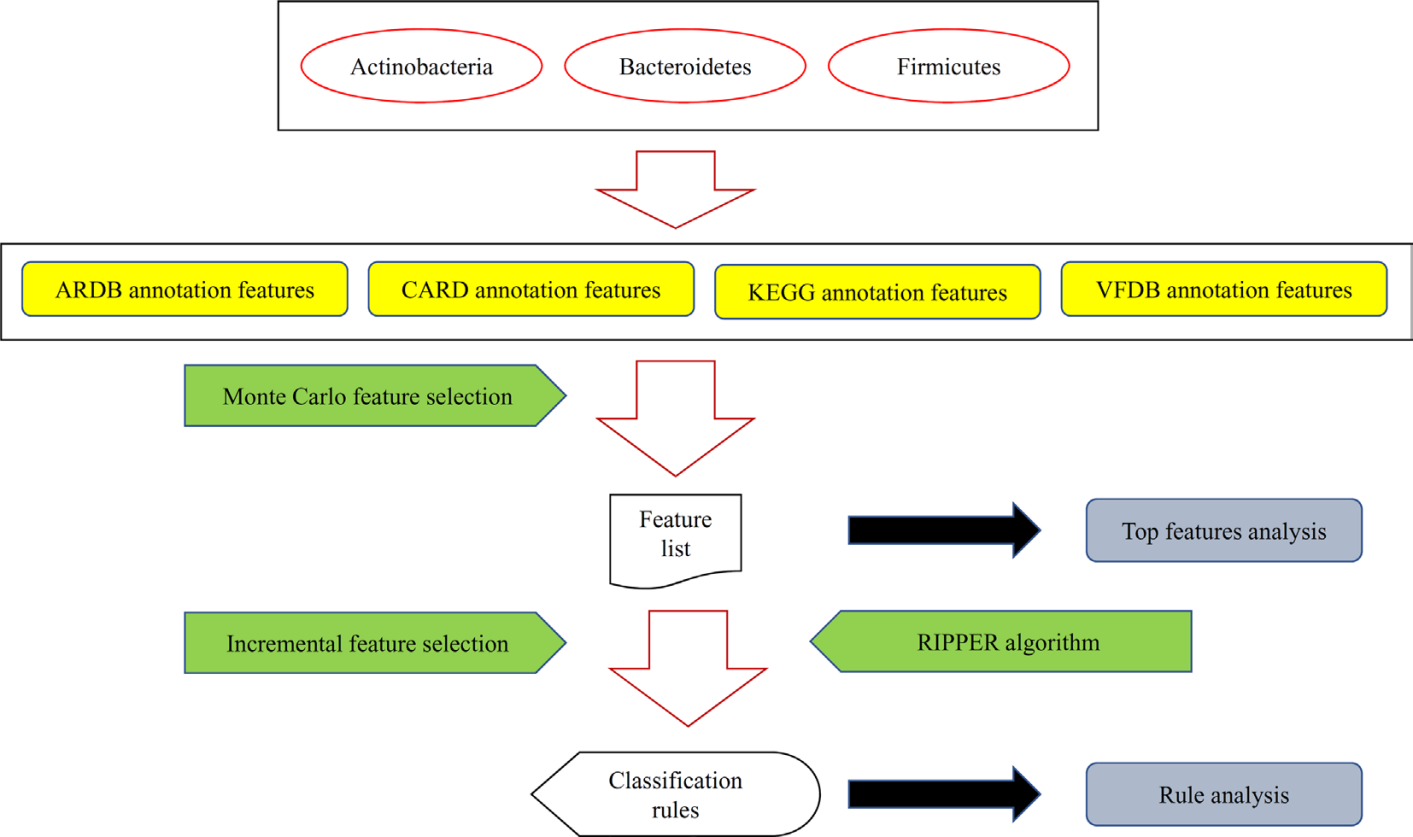
A flow chart to illustrate the procedures of identifying microbiota signature and functional rules for bacterial subtypes in human intestine. Bacteria in three human gut bacteria phyla were represented by four types of features. These features were analyzed by the Monte Carlo feature selection method, resulting in a feature list. For some top features, an extensive analysis was performed. Furthermore, the incremental feature selection method, incorporating the rule learning algorithm (RIPPER algorithm), was applied on the feature list to construct optimal classification rules, which were also extensively analyzed.

In this study, we extracted 993 features to represent each sample. These features consist of 342 ARDB features, 259 CARD features, 243 KEGG features, and 149 VFDB features, wherein the names and values are given in **Supplementary Table S1**. Then, several advanced computational methods were adopted to analyze these features. The entire procedures are illustrated in **Figure 1**. Clearly, not all features have the same importance for distinguishing samples from different bacterial groups; as such, the features are ranked and selected using the RI scores from MCFS. The RI scores of individual features are given in **Supplementary Table S2**. A total of 432 of all 993 features have RI scores larger than zero and thus have discriminated ability for different bacterial groups. Other features were discarded in the following analysis.

**Figure 2:**
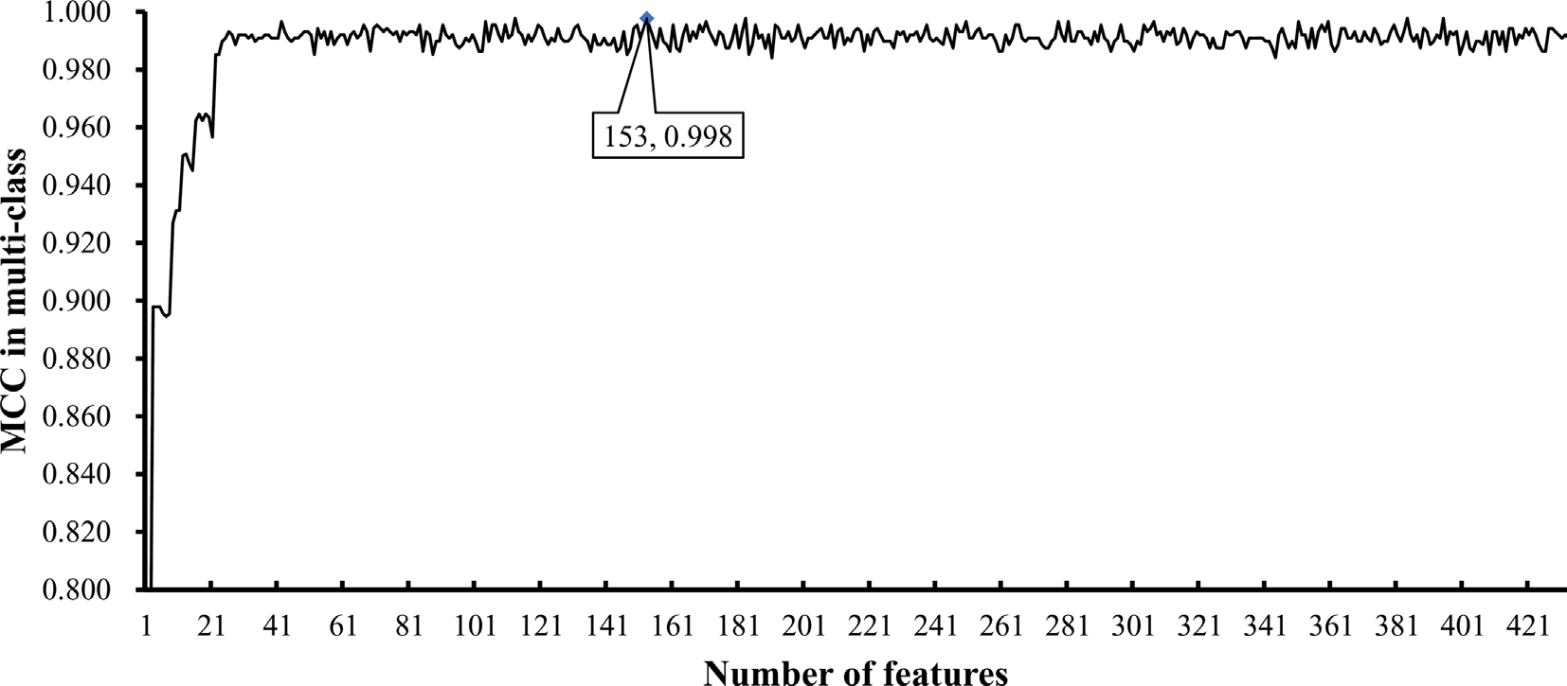
Optimal performance of IFS with RIPPER algorithm. The RIPPER algorithm provided the highest MCC (0.998) when top 153 features were used.

**Figure 3:**
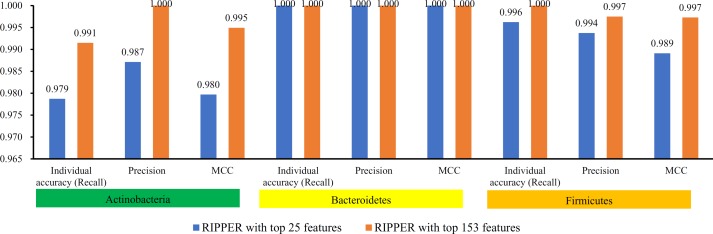
Performance of RIPPER algorithm with top 25 and 153 features on each phylum. The RIPPER algorithm with top 153 features provided nearly perfect classification, while the RIPPER algorithm yielded a little lower performance.

**Figure 4:**
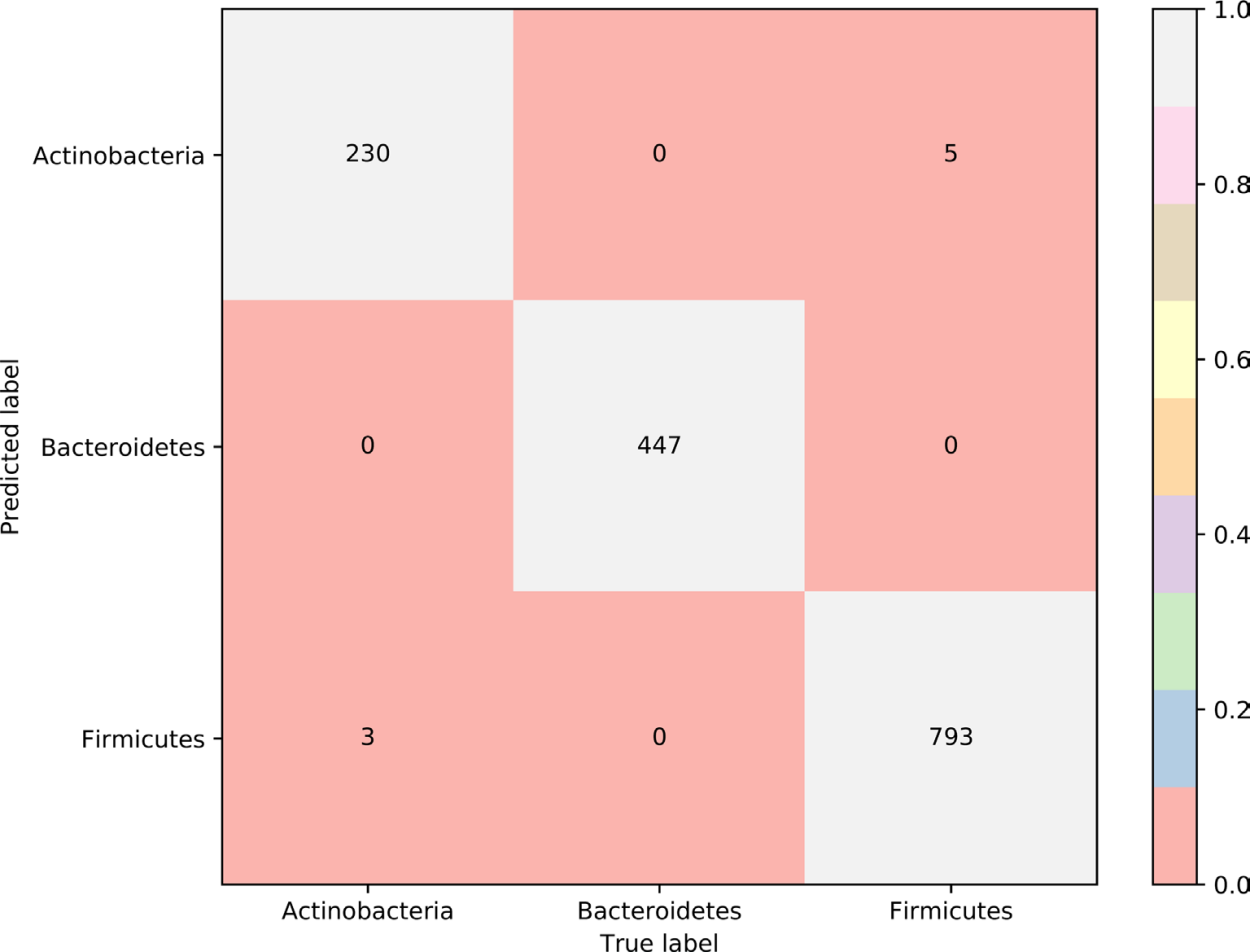
Confusion matrix yielded by the RIPPER algorithm with top 25 features. The accuracy of Bacteroidetes reached 1.000, while those of two other phyla were higher than 0.970, indicating the high performance of RIPPER algorithm with top 25 features.

To further select the optimum features from the 432 features, we used IFS with RIPPER for sample classification. RIPPER was trained and evaluated on the samples consisting of features from individual feature subsets by 10-fold cross-validation. As shown in **Figure 2**, among the top 432 features, the best MCC in multi-class of 0.998 and an overall accuracy of 0.999 were obtained when the top 153 features were used. The individual accuracy (recall), precision and MCC for each phylum are shown in **Figure 3**. It can be seen that each of these measurements was larger than 0.990, indicating the good performance of RIPPER on top 153 features. In particular, we obtained a high MCC in multi-class of 0.991 and an overall accuracy of 0.995 when only the top 25 features were used. The detailed predicted results were counted as a confusion map, as shown in **Figure 4**. Its performance on each phylum is shown in **Figure 3**, which was a little lower than that of the RIPPER with top 153 features; however, it was still very high. The corresponding performance of the RIPPER with the number of features ranging from 1 to 432 are shown in **Supplementary Table S3**. The results indicate that the interpretable rule-based method RIPPER is close to perfectly classify the samples from Actinobacteria, Bacteroidetes, and Firmicutes.

**Figure 5:**
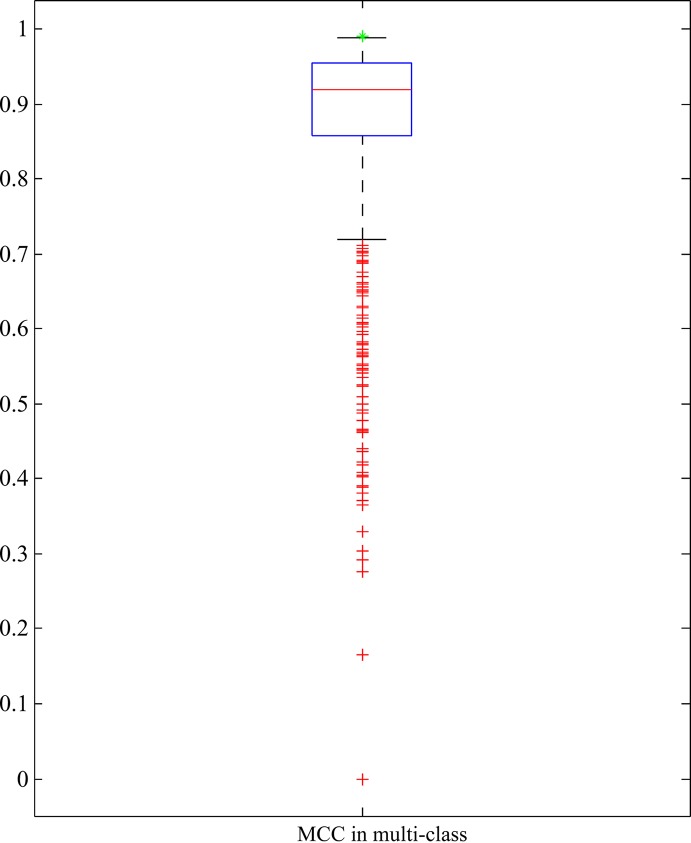
Box plot to show the performance of RIPPER algorithm with 25 features that are randomly selected from all features. The green star strands for the MCC in multi-class yielded by RIPPER algorithm with top 25 features, which is higher than all other MCCs in multi-class on randomly selected 25 features.

As mentioned above, RIPPER with top 25 features yielded quite high performance. To indicate the importance of these 25 features, we did the following test: 1000 feature subsets containing 25 features were randomly produced. RIPPER was trained on the samples represented by features from each of these feature subsets and evaluated by 10-fold cross-validation. Obtained MCCs in multi-class are illustrated in a box plot, as shown in **Figure 5**, in which the MCC in multi-class yielded by the RIPPER with top 25 features is also listed. It can be observed that all MCCs in multi-class on randomly produced feature subsets were lower than that yielded by the RIPPER with top 25 features. It is suggested that top 25 features were very important for identifying bacteria in different phyla. Therefore, we established five significant classification rules on all bacteria represented by top 25 features, as listed in **Table 1**, to elucidate how RIPPER can make accurate prediction. The details of these learned rules are discussed below. The results demonstrate the satisfactory discriminate powers of the five produced classification rules for different bacterial groups.

**Table 1:** Five classification rules produced by the RIPPER algorithm for Actinobacteria, Bacteroidetes, and Firmicutes.

Rules	Criteria	Bacteria group
Rule 1	Genetic Information Processing: Folding, sorting, and degradation: Proteasome > = 1	Actinobacteria
Rule 2	(Human Diseases: Drug resistance: Cationic antimicrobial peptide (CAMP) resistance < = 0) and (Genetic Information Processing: Folding, sorting, and degradation: Protein processing in endoplasmic reticulum > = 2)	Actinobacteria
Rule 3	(Cellular Processes: Transport and catabolism: Peroxisome < = 0) and (Genetic Information Processing: Folding, sorting, and degradation: Protein processing in endoplasmic reticulum > = 2) and (Human Diseases: Drug resistance: Cationic antimicrobial peptide (CAMP) resistance < = 1)	Actinobacteria
Rule 4	Organismal Systems: Digestive system: Protein digestion and absorption > = 1	Bacteroidetes
Rule 5	others	Firmicutes

## Discussion

In this study, we attempted to integrate different feature sets from ARGD (Liu and Pop, 2009), CARD (McArthur et al., 2013; Jia et al., 2017), VFDB (Liu et al., 2019), and KEGG (Kanehisa, 2002; Tanabe and Kanehisa, 2012) databases. Basing on these collective features and original datasets, we accurately distinguished the common gut bacteria into three major clusters: Actinobacteria, Bacteroidetes, and Firmicutes. We not only identified the crucial features from the four known datasets that contributed most to such clustering but also set up a novel quantitative rule set for the accurate clustering of gut bacteria. All the predicted results (i.e., features and rules) were supported by solid experimental evidence presented in literature. We screened the top features and rules in our optimal prediction list for further discussion and analyses below due to page limitation.

### Analysis of Optimal Features for Subtyping of Gut Bacteria

Using machine learning models, we screened a group of proper features to distinguish three common gut bacterial subgroups. The first significant distinctive feature (F_740) is a metabolism describing feature: glycan biosynthesis and lipopolysaccharide biosynthesis associated metabolism. According to recent publications, bacteria from Actinobacteria (King et al., 2009; Alshalchi and Anderson, 2015), Bacteroidetes (Jacobson et al., 2018), and Firmicutes (d'Hennezel et al., 2017) participate in these biological processes. In contrast to Actinobacteria and Bacteroidetes, Firmicutes directly promotes the biosynthesis of lipids and contributes to the pathogenesis of obesity (d'Hennezel et al., 2017). The activation of such metabolic processes was finally decided by the relative abundance of Firmicutes compared with the other bacterial phyla. Therefore, F_740 could be a novel and effective feature for subtyping different bacterial subgroups.

The following feature marked as F_602 describes cell growth and death-associated processes, including apoptosis. In general, the balance between cell growth and death in the intestine is usually regulated and maintained by inflammatory reactions (Neurath et al., 1998; Pickard et al., 2017) and lipopolysaccharide production (Guo et al., 2013). The production of lipopolysaccharides is significant for the survival of gut cells. According to recent publications, lipopolysaccharide production is correlated with the relative abundance ratio between Bacteroidetes and Firmicutes (Jeong et al., 2015; Kim et al., 2016). Therefore, the stable status of cell growth and death-associated processes may be sufficiently effective and sensitive for evaluating the relative abundance of such two major bacterial subtypes, thereby validating the efficacy of our new method.

F_823 describes the general protein digestion and absorption processes of the digestive system, and different bacterial subgroups play different roles in the digestion and absorption of different nutrients (Flint et al., 2012; Valdes et al., 2018). For example, the digestion and absorption of lipids and proteins as a proper instance again; as such, different subgroups of bacteria contribute differently to such processes. In contrast to fat metabolism, a case of protein metabolism, the high abundance of bacterial subgroups, such as Bacteroidetes, indicates the high activation status of protein digestion and absorption (Turnbaugh et al., 2006). Therefore, F_823, as an indicator of the activity degree of protein metabolism, may contribute to the distinction of different bacterial subgroups.

F_608, as a complicated feature describing the formation of biofilm, was screened to distinguish different gut bacterial subgroups. In 2015, a systematic review on microbial biofilms and associated gut diseases confirmed that the abundances of Firmicutes and Bacteroidetes rather than that of Actinobacteria are functionally related to biofilm. The relative contributions of the three clusters of gut bacteria on biofilm regulation would be quite different (von Rosenvinge et al., 2013). Therefore, the biological characteristics of gut biofilm may also be a potential biomarker for the distinction of different bacteria subgroups.

The finally discussed high-ranked feature, named as F_756, describes the biosynthesis of steroid hormone. In 2013, a review on gut microbiome summarized the specific role of steroid hormones in the interactions between the gut bacteria and host humans (Garcia-Gomez et al., 2013). According to this review, only bacteria from clusters such as Actinobacteria, Proteobacteria, and Firmicutes were confirmed to participate in the biosynthesis and metabolism of steroid hormone to date. However, Bacteroidetes does not. In addition, the dominant phyla, such as Actinobacteria and Firmicutes, can express hydroxysteroid dehydrogenase; this phenomenon is essential for steroid hormone metabolism (Kisiela et al., 2012). Therefore, such feature has significant functional importance for bacterial subgrouping.

### Analysis of the Optimal Rules for Gut Bacteria Subtyping

The use of our newly presented computational approaches to determine the optimal features has been validated by recent publications. Apart from such qualitative analysis results, quantitative analysis was performed to distinguish different bacterial subgroups. Based on Jrip algorithm, also known as the RIPPER algorithm, we identified five effective rules for explaining the distinction of bacterial subgroups.

The first rule contains one feature describing the biological processes of proteasomes involving folding, sorting, and degradation of functional proteins. According to recent publications, proteasomes are self-compartmentalized proteolytic organelles only identified in Archaea, Actinobacteria, and eukaryotes but not in Bacteroidetes or Firmicutes (Valas and Bourne, 2008; Ziemski et al., 2018). Therefore, regarding such feature as a quantitative parameter for the identification of Actinobacteria is quite reasonable.

The next rule indicates cationic antimicrobial peptide (CAMP) resistance (F12) and protein folding in the endoplasmic reticulum as another two quantitative parameters for the recognition of Actinobacteria subgroup. According to recent reports, cationic antimicrobial peptides mediate the bacterial resistance against most Actinobacteria and Firmicutes (Anaya-Lopez et al., 2013). Therefore, the first parameter may distinguish Actinobacteria and Firmicutes from other bacterial subgroups. As for the next parameter, Actinobacteria has a specific structure called peroxisomes, sharing similar biological functions with the endoplasmic reticulum (Duhita et al., 2010; Gabaldon and Capella-Gutierrez, 2010). Therefore, the combination of the two parameters refers to the accurate identification of Actinobacteria, thereby validating the efficacy and accuracy of our prediction.

Next, the third rule has three parameters involved in protein modification. Apart from parameters F24 and F12, the effective parameter F7 describes the transport and catabolism of peroxisomes, which were identified and discussed to be unique in Actinobacteria, thereby validating our prediction (Duhita et al., 2010; Gabaldon and Capella-Gutierrez, 2010).

The fourth rule is associated with the differential performance of the general protein digestion and absorption processes of the digestive system with different distribution patterns of bacteria. The high activation status of protein digestion and absorption pattern in the gut indicate the abundance of Bacteroidetes (Turnbaugh et al., 2006), corresponding with our rules.

Overall, all optimal features and rules for the distinction of different bacterial subgroups are accurate and efficient with solid publication supports. The accurate clustering of gut bacteria is the foundation for microbiome studies of the human intestine. For a long time, applying microbiome clustering based on sequencing data is difficult and time consuming due to the complicated described feature sets. Here, with the help of machine learning models, we identified the core features for microbiome distinction and set up a group of accurate distinctive rules for explaining such clustering problem. Therefore, using proper machine learning models, the present study reveals an accurate and elaborate panorama for gut microbe and provides a novel tool for further studies on the microbiome.

## Data Availability Statement

Publicly available datasets were analyzed in this study. This data can be found here: https://db.cngb.org/search/project/CNP0000126/.

## Author Contributions

All authors contributed to the research and reviewed the manuscript. LC and YZ designed the study. LC and DL performed the experiments. YS, HW, and YL analyzed the results. LC wrote the manuscript.

## Funding

This work was supported by grants from the Science and Technology Research Projects of Anhui Province (201904b11020043, 201904e01020014), Natural Science Foundation of Anhui Province (1808085MC60), and the National Key R&D Program of China (2018YFD1100104).

## Conflict of Interest

The authors declare that the research was conducted in the absence of any commercial or financial relationships that could be construed as a potential conflict of interest.

## Supplementary Material

Supplementary Table S1 Data matrix of analysis.

Supplementary Table S2 Top features with their importance scores calculated by the MCFS.

Supplementary Table S3 Ten-fold cross-validation performance of IFS with RIPPER algorithm.
